# *In vitro* activity of *Alkanna frigida* extracts in comparison with glucantime against *Leishmania major*

**Published:** 2013-06

**Authors:** Fariba Jafari, Abbasali Nourian, Asghar Fazaeli, Alireza Yazdinezhad, Ali Haniloo

**Affiliations:** 1 Department of Medical Parasitoloy and Mycology, Medical School, Zanjan University of Medical Sciences and Health Services; 2 Department of Pharmacognozy, Faculty of Pharmacy. Zanjan University of Medical Sciences and Health Services, Zanjan, Iran

**Keywords:** *Alkanna frigida*, *In vitro* activity, *Leishmania major*, cutaneous leishmaniasis

## Abstract

**Background and Objectives:**

Current chemotherapies of cutaneous leihmaniasis have faced to some problems and limitations; Development of new leishmanicidal drugs from different sources like herbal plants, are crucially important. The objective of the present study was evaluation of *in vitro* activity of *Alkanna frigida* extracts in comparison with glucantime against *Leishmania major*.

**Materials and Methods:**

*L. major* promastigotes were exposed to different concentrations of the *A. frigida* extracts, processed by ethyl acetate, ethanol, hexane and chloroform. The inhibitory effect, as the IC_50_, were calculated after 24, 48 and 72 hours by linear regression analysis values of the concentrations employed.

**Results:**

The significant inhibition was observed after 24 and 48 hours with different concentrations of compounds (p < 0.05 in all tests). All extracts had potent activity against proliferation of the promastigotes, comparing to the untreated negative control. It could compete with the glucantime efficacy in some concentrations. Ethyl acetate and ethanol extractions showed potent IC_50_ value, 106 µg/ml and 86 µg/ml, respectively. Hexane and chloroform extractions had poor efficacy after 24 hours; however, the efficacy increased after 48 and 72 hours.

**Conclusion:**

The results indicated that the *A. frigida* has appropriate inhibitory effects on the growth of *L. major* promastigotes *in vitro* and can be of herbal targets for further investigation *in vivo*.

## INTRODUCTION

Leishmaniasis is one of the most significant health problems amongst a collection of neglected tropical and sub-tropical diseases. It is caused by different species of the genus *Leishmania*, which are transmitted by dipterans of the genera *Phlebotomus* in the old World and *Lutzomyia* in the new World. Based on the world health organization (WHO) reports in past years, approximately, 12 million individuals were estimated to be infected and 350 million people were at risk of leishmaniasis in many countries (1). Annual global incidence of cutaneous leishmaniasis, based on recently reports from endemic countries, is estimated to rate between 0.7 to 1.2 million cases (2). *Leishmania major* and *L. tropica*, the two causative agents of cutaneous leishmaniasis in Iran, have long been endemic in several definite foci, affecting tens of thousands of people (3). Emergence of the disease in additional new foci in this country have been reported in recent years (4–8).

Treatment of Leishmaniasis has been a big challenge since a prolonged therapy is needed. Relapses of the infection and differential susceptibilities between taxonomically closely related species of *Leishmania* are also of major problems (9). Contraversial challenge is also the case for antileishmanial drugs efficacy (10) even for the same clinical type of leishmaniasis. The first choice of drugs, used for treatment of leishmaniasis, are pentavalent antimonial compounds (meglumine antimoniate/glucantime and sodium stibogluconate); while amphotericin B and pentamidine are used as the second-line chemotherapy. Despite the current application of these compounds, they are restricted by side effects to the patients, i.e. anorexia, vomiting and nausea (9). Drug resistance of the Leishmania parasites against some compounds, such as the above mentioned traditional drugs is also reported (11, 12). Several other compounds have been applied for the healing of cutaneous leishmaniasis which are reviewed by some researchers (13, 14). Although, leishmanicidal drugs originated from different sources like synthetic compounds, natural products extracted from plants and marine sources have shown different degrees of efficacy in the treatment of experimental different related leishmaniasis, so far, there is no standard therapy available. One of the reasons could be unfavorable results in animal experiments, and only a small published series of susceptibility testing. Generally, available data have shown that a wide range of plant families and species have trypanocidal and leishmanicidal activities (15–17). Natural compounds may be advantageous to the chemical products for treatment of the disease for several reasons, i.e. the side effects of chemical compounds. *Alkanna* is described as a genus of herbaceous plants, having around 60 species, in the family of Boraginaceae. The original *Alkanna* plant is a native of the Levant but is now found wild and cultivated, throughout the Europe, around the Mediterranean and the Middle East. *Alkanna tinctoria* grows in the south of France with antibacterial activity which is often used to improve acute inflammatory, swollen, and burned wounds (18). *Alkanna* compounds have revealed activity and effectiveness on the treatment of acute inflammatory lesions and severe victim burns (19). *Alkanna frigida* extract was also reported to have potential anti-inflammatory and anti-nociceptive effects in experimentally affected laboratory animals (20).

Based on the above evidences indicating the potential remedial effects in this herbal genus, we anticipated that the species *A. frigida* could be a good candidate to be examined as a possible antileishmanial plant. This species is broadly distributed in North and North West of Iran and some neighboring countries like Iraq. The objective of the present study was evaluation of *in vitro* activity of *A. frigida* extracts, processed by ethyl acetate, ethanol, hexane and chloroform, in comparison with glucantime (meglumine antimoniate) as an effective (positive) control, against promastigotes of *L. major*. Glucantime is proven to be effective against *L. major* and can stop the promastigote growth *in vitro*.

## MATERIALS AND METHODS

### Alkanna frigida extraction and purification

*A. frigida* plant is extremely available on Hashtkhani Mountain (2700-2900 meters of altitude) in the North West of Iran and they were collected before flowering stage at the beginning of June 2011. The voucher specimen was identified and deposited at the Herbarium of Department of Pharmacognozy, Faculty of Pharmacy, Zanjan University of Medical Sciences, Iran (Voucher Number: ZUMS-1027). The plant source and the species was the same as what was previously confirmed by Dr Gholamreza Amin as referred previously (21). The plant extraction was performed based on Harborne, 1998, (22) using percolation and rota-vapor apparatuses. Briefly, the root limb parts of the *A. frigida* were dried in shadow at 25°C for 3 days and stored at 4°C until use. Based on the percolation method, a portion of 200gr of *A. frigida* root was extracted by using 200 ml of hexane, and consequently with chloroform, ethyl acetate and 80% ethanol (22). 1% DMSO in RPMI-1640 medium was used for dissolving the crude extracts.

*Parasite Strain and Culture: Leishmania major* promastigotes (MRHO/IR175/ER) were obtained from Centre for Training and Research in Skin Diseases and Leprocy, Tehran University of Medical Sciences. They were cultivated *in vitro* in RMPI-1640 medium (GIBCO) containing 10% inactivated foetal bovine serum (FBS) (GIBCO) for preparation of adequate promastigotes, then incubated in a standard air atmosphere at 26°C in roux flasks.

### *In vitro* antileishmanial activity testing

The promastigote forms of *L. major* were collected in the exponential phase and inoculated in the 96 well microplates to a final concentration of 3 × 10^6^ parasites per well and kept at 26°C. The plant extracts obtained by ethanol, ethyl acetate, chloroform, and hexane, were dissolved in RPMI medium, at dosages of 62.5, 125, 250 and 500 µg/ml. All tests were done in triplicate. The effects of each compound against promastigote forms were tested at 24, 48, and 72 hours after the exposure. Live parasites were counted using a Neubauer haemocytometric chamber under a microscope. Alongside of the plant extracts, glucantime with the concentrations of 62.5, 125, 250 and 500 µg/ml was used as the effective positive control and untreated promastigote cultures were used as negative control. The inhibitory effects of the obtained compounds on the Leishmania promastigotes in comparison with those of glucantime were calculated in relation to negative control (untreated cultures) using the following formulae: PI = (PC-PT)/PC × 100 where PI is the percentage of inhibition, PC is the number of live amastigotes in the control (absence of the plant extracts) and PT is the number of amastigotes in the drug-treated culture wells.

**Fig. 1:**
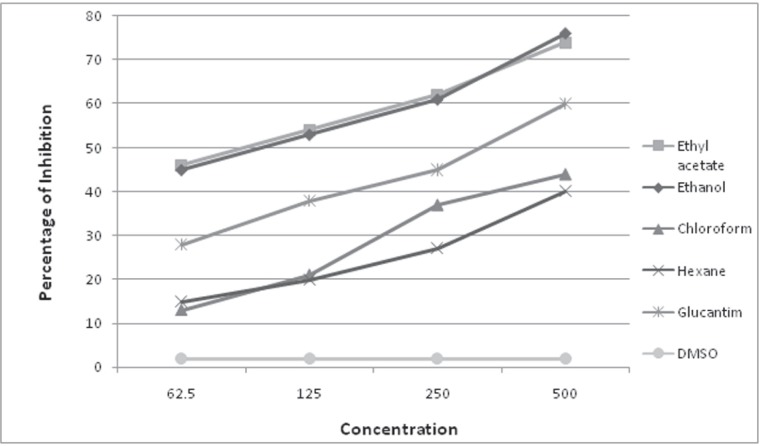
Inhibitory effects of different concentrations of *Alkanna frigida* extracts (µg/ml), compared to glucantime, on *Leishmania major* promastigotes in RPMI-1640 after 24 hrs.

The IC_50_, which is the concentration required to inhibit 50% growth of promastigotes after 24 hours, was calculated by linear regression analysis; the percentages of the promastigote death versus different concentrations of the plant extracts were submitted to the Excel software, analysis of which resulted in calculation of the IC_50_ which is mentioned in Fig. 1.

## RESULTS

The inhibitory activities of *A. frigida* extracts on growth of *L. major* promastigotes are summarized in Table 1. Significant inhibition of parasite growth was observed after 24 and 48 hours of treatment with different concentration of compounds. Glucantime was used for the comparison as a reference drug. The results showed that the extracts had potent activity against proliferation of the promastigotes. The inhibitory effects of ethyl acetate, ethanolic, chloroform and hexane extracts at the lowest concentration (62.5 µg/ml), after 24 hours were 46%, 45%, 13% and 15%, respectively (Table 1). In these conditions, it was 28% for glucantime. Parasite count and observation under a dark field microscope, showed appropriate growth and viability of promastigotes in the negative control (RPMI-1640 with 1% DMSO, without drug); the parasite death was maximum 2%. Ethyl acetate and ethanol extracts showed potent IC_50_ value, 106 µg/ml and 86 µg/ml, respectively; whereas hexane and chloroform extracts had poor efficacy on growth of parasite after 24 hours (Fig. 1). In contrast, significant results have been observed after 48 and 72 hours, IC_50_ value for chloroform extract was 330 µg/ml and 68 µg/ml for 48 and 72 hours, respectively. In addition, hexane extract IC_50_ had potent activity (384 µg/ml for 48 hr) and (98 µg/ml 72 hr). IC_50_ value for reference drug (glucantime) was 340 µg/ml increased in efficacy and showed a significant difference in comparisons with the examined compounds. Ethanol and ethyl acetate with the concentration of 500 µg/ml inhibited 76% and 74% of parasite, while glucantime in the same concentration had 60% elimination (Table 1).

**Table 1:** Inhibitory effects of ethanolic, ethyl acetate, chloroform and hexane extracts of *Alkanna frigida* on *Leishmania major* promastigotes *in vitro*.

The percentages of inhibition																

Concentration µg/ml	Ethyl acetate			Ethanol			Chloroform			Hexane			Glucantime			*P value* Chi-squar

	24h*	48h	72h	24h	48h	72 h	24 h	48 h	72 h	24 h	48 h	72h	24h	48h	72h	
62.5	46	57	68	45	59	75	13	30	46	15	33	48	28	47	57	0.00001
125	54	63	72	53	64	79	21	38	56	20	41	55	38	52	65	0.00001
250	62	72	77	61	70	87	37	45	62	27	46	60	45	58	75	0.00001
500	74	84	87	76	82	96	44	61	72	40	54	68	60	70	81	0.00001

* h (hours), indicate the post treatment times of the inhibition calculations.

By using regression model and estimation of the relative danger in comparison (Table 2), we found that by passing the time, the effects of ethanol and ethyl acetate extracts of *A. frigida* plant could get closer to the effects of glucantime drug (Tables 2–4). During 72 hours the relative danger of ethyl acetate extract was equal to 1.09 and that of ethanol extract was equal to 1.21 (Table 4).

**Table 2:** Poason regression for investigation of the antileishmanial effects of ethanolic, ethyl acetate, choloroform, and hexane extracts of *Alkanna frigida* plant after 24 hours.

Chemical extracts	Upper Bound	Lower Bound	Z	Relative danger	estimation	P value (Chi-square)
Ethyl acetate	0.323	0.321	555.649	1.37	0.322	0.00001
Ethanol	0.319	0.317	547.836	1.37	0.318	0.00001
Chloroform	-0.395	-0.398	-.569.799	0.67	-0.397	0.00001
Hexane	-0.515	-0.518	-715.337	0.59	-0.517	0.00001
Glucantime (compared group)				1.0		

**Table 3:** Poason regression for investigation of the antileishmanial effects of ethanolic, ethyl acetate, choloroform, and hexane extracts of *Alkanna frigida* plant after 48 hours.

Chemical extracts	Upper Bound	Lower Bound	Z	Relative danger	estimation	P value (Chi-square)
Ethyl acetate	0.90	0.88	186.607	1.09	0.89	0.00001
Ethanol	0.193	0.192	411.458	1.21	0.192	0.00001
Chloroform	-0.163	-0.165	-.320.518	0.84	-0.164	0.00001
Hexane	-0.184	-0.186	-.360.327	0.83	-0.185	0.00001
Glucantime (compared group)				1.0		

**Table 4:** Poason regression for investigation of the antileishmanial effects of ethanolic, ethyl acetate, choloroform, and hexane extracts of *Alkanna frigida* plant after 72 hours.

Chemical extracts	Upper Bound	Lower Bound	Z	Relative danger	estimation	P value (Chi-square)
Ethyl acetate	.196	.194	377.838	1.2153	.195	0.00001
Ethanol	.193	.191	370.516	1.2116	.192	0.00001
Chloroform	-.265	-.267	-457.133	0.76	-.266	0.00001
Hexane	-.265	-.267	-457.133	0.76	-.266	0.00001
Glucantime (compared group)				1.0		

## DISCUSSION

The results showed that *Alkanna frigida* extracts have potent activity against proliferation of *L. major* promastigotes. Ethyl acetate and ethanol extracts showed the IC_50_ values of 106 µg/ml and 86 µg/ml, respectively. Hexane and chloroform extracts had poor efficacy after 24 hours; however, the efficacy increased after 48 and 72 hours.

Although the agents of leishmaniasis are susceptible to most antileishmanial drugs *in vitro*, treatment may be a big challenge because of the disease relapses due to diverse susceptibility to the current chemotherapies, within the species and/or isolates of the causative *Leishmania* parasites. Meglumine antimoniate (glucantime) and sodium stibogluconate (pentostam) are in the first line of therapy; amphotericin B with severe side effects and insufficient improvement in many reported cases are used as the second-line chemotherapy (15). Moreover, in some severe reported cases in the literature, cutaneous leishmaniasis showed failure therapy, and consequently drug does not seem to be the optimal choice for the treatment of this type of infection. Therefore, plants can be valuable basic sources for development of new therapeutic compounds. *Alkanna* plants and their derivatives are among the most attractive plant derivatives that might enrich the current therapy options, due to their extremely large range of biological properties (18). Thus developing newly effective drugs on the treatment of the cutaneous leishmaniasis are extremely considered. Common treatment is using pentavalent antymonials which is often toxic and can lead to the emergence of some side effects. Since long time ago, researchers have focused on the herbal drugs. In 1976, Parpagergiou et al, showed that *Alkanna tinctoria* extract has antibacterial effects and also healing effect on wound (18). Also, *Alkanna tincktoia* extract on the wounds of burning was conducted on the rabbits and the results show that the plant extract has positive effects on the wounds of burning (23). In a study by Esfahani *et al*. (2012), potential anti-inflammatory and anti-nociceptive effects of ethanolic extract of *Alkanna frigida* in rat and mouse inoculated by carrageenan and formalin, were also reported (20). They found significant decrease in carrageenan induced inflammation using 200 and 400 mg/kg of the extract after 1 and 2h; remarkable anti-nociceptive effect was also of their findings. In our study, leishmanicidal activity of *A. frigida* plant on *L. major* promastigotes, revealed that ethyl acetate extract with a concentration of 106 µg/ml during 24 hours can inhibit 50% of promastigotes propagation. The leishmanicidal activity of ethanol extract was close to that of ethyl acetate (Table 1, Fig. 1). However, chloroform and hexane extracts showed lower efficacy in comparison with ethanol and ethyl acetate extracts. The ethyl acetate and ethanol extracts after 48 and 72 hours also showed to have the most effective leishmanicidal activity in comparison with the other two chemical extracts. The estimation of relative risk also indicated that the antileishmanial effects of these two extracts, are close and even higher than the effects of glucantime (Tables 2–4). Similarly, in a study of leishmanicidal evaluation of the *Artemisia* species by Imami and colleagues in 2008, the ethanol extract was also more effective than the other extracts, including chloroform and hexane extracts (24). Alcoholic extract of another plant, *Calendula officinalis*, on Leishmania promastigotes was more effective, comparing to its watery extracts (25). Different concentrations of the ethanol and ethyl acetate extracts in comparison with glucantime under the same conditions, exhibited higher efficacy (Table 1, Fig. 1). However, based on these data, the *A. frigida* extracts cannot be considered superior to glucantime and remain to be evaluated further, in terms of their toxicity effect and compound analysis. It was basically concluded that the *A. frigida* extracts have inhibitory effects on the *L. major* promastigote proliferation and the ethanol and ethyl acetate extracts have more leishmanicidal effects than those of chloroform and hexane extracts.
